# A multiscale model of early cell lineage specification including cell division

**DOI:** 10.1038/s41540-017-0017-0

**Published:** 2017-06-09

**Authors:** Alen Tosenberger, Didier Gonze, Sylvain Bessonnard, Michel Cohen-Tannoudji, Claire Chazaud, Geneviève Dupont

**Affiliations:** 1 Unité de Chronobiologie Théorique, Faculté des Sciences, Université Libre de Bruxelles (ULB), Brussels, Belgium; 2 Unité de Génétique Fonctionnelle de la Souris, UMR3738 CNRS/Institut Pasteur, Paris, France; 3 Clermont Université, Laboratoire GReD, Clermont Université, Université d’Auvergne, Clermont-Ferrand, France; 4 Inserm, UMR1103, Clermont-Ferrand, France; 5 CNRS, UMR6293, Clermont-Ferrand, France

## Abstract

Embryonic development is a self-organised process during which cells divide, interact, change fate according to a complex gene regulatory network and organise themselves in a three-dimensional space. Here, we model this complex dynamic phenomenon in the context of the acquisition of epiblast and primitive endoderm identities within the inner cell mass of the preimplantation embryo in the mouse. The multiscale model describes cell division and interactions between cells, as well as biochemical reactions inside each individual cell and in the extracellular matrix. The computational results first confirm that the previously proposed mechanism by which extra-cellular signalling allows cells to select the appropriate fate in a tristable regulatory network is robust when considering a realistic framework involving cell division and three-dimensional interactions. The simulations recapitulate a variety of *in vivo* observations on wild-type and mutant embryos and suggest that the gene regulatory network confers differential plasticity to the different cell fates. A detailed analysis of the specification process emphasizes that developmental transitions and the salt-and-pepper patterning of epiblast and primitive endoderm cells from a homogenous population of inner cell mass cells arise from the interplay between the internal gene regulatory network and extracellular signalling by Fgf4. Importantly, noise is necessary to create some initial heterogeneity in the specification process. The simulations suggest that initial cell-to-cell differences originating from slight inhomogeneities in extracellular Fgf4 signalling, in possible combination with slightly different concentrations of the key transcription factors between daughter cells, are able to break the original symmetry and are amplified in a flexible and self-regulated manner until the blastocyst stage.

## Author summary

The early development of the mammalian embryo involves cell divisions and highly regulated lineage specification events. Cell fates are determined by gene regulatory networks exhibiting multiple steady states. The question arises as to how these networks interact with extracellular signalling, cell division and cell movement. Here, we investigate this question in the context of establishment of the salt-and-pepper pattern of epiblast (Epi) and primitive endoderm (PrE) cells within the inner cell mass (ICM) of the preimplantation embryo in the mouse, using a multi-scale computational model. The three cell fates correspond to three stable steady states of the gene regulatory network, which coexist in the salt-and-pepper pattern. The specification process is self-regulated through extracellular signalling and is robust towards cell division and cell movement.

## Introduction

The development of the single mammalian cell zygote into an embryo arises through the combined effect of cell divisions and differentiations. Until the blastocyst stage, two specifications occur. The first one, taking place at the eight cell stage, gives rise to the inner cell mass (ICM) and the trophectoderm (TE). The second one corresponds to the specification of ICM cells into cells of the epiblast (Epi) and of the primitive endoderm (PrE). Among these three cell types, pluripotent Epi cells will give rise to the embryo itself, whereas TE and PrE cells form extra-embryonic structures such as placenta.^1–4^

The lineage specification of ICM cells into Epi and PrE cells is tightly regulated by a gene regulatory network (GRN) and by inter-cellular signalling. Nanog and Gata6, two antagonistic factors, have a key role in this process as Nanog is necessary to produce Epi cells,^5, 6^ and Gata6 is required for the specification of PrE cells.^7, 8^ In mice, from the 8-cell stage corresponding to the embryonic day ~E2.25 to the 32-cell stage (E3.25), Nanog and Gata6 proteins are coexpressed at increasing levels in almost all ICM cells.^9^ Then, from this stage, their expression patterns start to become mutually exclusive and at E3.75, Epi and PrE cells, expressing Nanog and Gata6, respectively, constitute two different cell populations that are arranged in a salt-and-pepper pattern.^9, 10^ In a later stage, cells rearrange in such a way that PrE cells form an epithelium that separates the Epi cells from the blastocoel.^11, 12^ The Epi/PrE fate choice is modulated by the Fgf/Erk signalling pathway. The specification of PrE indeed requires the expression of the Fgf receptor, *Fgfr2*, of its ligand Fgf4 and of the Erk adaptor *Grb2*.^11, 13–15^ Moreover, exogenous manipulations of the Fgf/Erk signalling pathway between E2.5 and E4.5 can force ICM cells to specify into a given fate: recombinant Fgf4 forces nearly all cells to adopt the PrE fate, whereas inhibitors of Fgf/Erk signalling largely favour the appearance of Epi cells.^16–18^

The process of blastocyst formation is a paradigm of self-organization, which takes place independently of the maternal environment. Owing to the complex network of interactions that are at play, computational approaches are very useful to back-up experimental investigations.^19^ Models have early emphasized that cell specification is best described as an evolution towards one or the other steady-state of a system that displays multiple steady states.^20–22^ It is known that a system based on two cross-inhibiting compounds, such as Nanog and Gata6, allows for bistability.^23^ Later studies demonstrated that such a system can also generate tristability if the expression of each of the two proteins can occur through an auto-activated pathway, in addition to the cross-inhibition pathway.^24, 25^ In a model devoted to the analysis of the specification of Epi and PrE cells from ICM cells in which Nanog and Gata6 expressions are controlled by both auto-activation and cross-inhibition (multiplicative terms instead of additive ones as in ref. 24), we showed that tristability can arise from the interplay between Fgf4 and the Nanog-Gata6 regulations. This model allowed the replication and prediction of a variety of cell behaviours observed in different conditions.^7, 26^

In this previous approach, we focussed on a static population of 25 cells. This simplified model did not consider the full systemic interactions between the dividing cells, the extracellular environment and the internal GRN. We have developed a three-dimensional (3D) multiscale model describing the interplay between cell division, signalling and gene expression, which is inherent to embryo development. We also consider that ICM cells are generated by two successive rounds of differentiative cell division. A detailed analysis of the behaviour of the model allows to recover experimental observations, to clarify underlying regulatory mechanisms and to collect data that are not, or hardly, attainable experimentally.

## Model development

### Modelling cell movement and cell division

We have developed a multi-scale model to describe early embryogenesis focusing on the emergence of Epi and PrE cells, and the formation of the salt-and-pepper pattern in mice. On the macroscopic scale, we describe cell division and interactions between cells, whereas on the microscopic scale, we consider biochemical reactions inside each individual cell and in the extracellular matrix. We use a spherical cell model, in which we track, for each cell, its volume, mass and the position of its centre of mass. All mechanical interactions between cells are modelled through pair-wise forces,^27, 28^ as described in Supplementary Information (Section 2). In addition, we compute the time evolution of the concentrations of key proteins in each individual cell using the gene regulatory network model developed in the next section.

Starting from the precocious ICM state, cells go through periodic waves of cell division. Each cell divides at a moment chosen randomly in the interval [(*n*−*δ*)*τ*,*nτ*], where *n* denotes the *n*th wave of cell division and *δ* is a parameter between 0 and 1 accounting for a possible asynchrony in individual division times. Default values for *τ* and *δ* are 12 h and ~40 min, respectively (Supplementary Table S2). At the moment of division, the mother cell is replaced by two daughter cells. The mass and the volume of each daughter cell are equal to one half of the mass and the volume of the mother cell. The plane of division is chosen randomly for each cell division and the two daughter cells are placed at opposite sides of the division plane (a more detailed description is given in Supplementary Information, Section 3). In most simulations, after the division the daughter cells inherit the values of the variables that characterise the regulatory network of their mother cell, which corresponds to an equal repartition of all compounds unless stated otherwise. To test the possible effect of an uneven repartition of molecules at division in some simulations, we introduce a parameter *η*_*i*_ such that the concentrations *C* of any compound in cell *i* is given by:

$${C_i}\left( {daughter} \right) = (1 \pm {\eta _i}) \cdot {C_i}\left( {mother} \right) \quad with \quad {\eta _i}\; \in \,\left[ {0,\,\eta } \right].$$

The change is opposite in each daughter cell to ensure the conservation of the number of molecules.

### Two waves of divisions leading to ICM cells

**Fig. 1:**
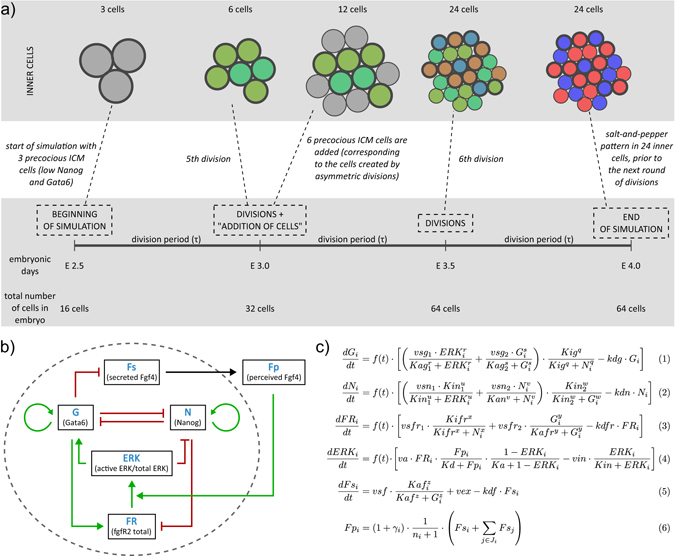
**a** Scheme of the model of development of epiblast (*Epi*) and primitive endoderm (*PrE*) lineage from inner cells (*ICM*). The model does not consider trophectoderm explicitly and starts from three inner cells originating from the first round of differentiative divisions (*In1*). The three cells begin the process of specification to Epi and PrE cells. In the second round of divisions, the three In1 cells divide into six cells, whereas differentiative divisions add six ICM cells (*In2*). Thus, at E3.0 the inner part of the embryo consists of six In1 and six In2 cells. The development of Epi and PrE lineage and the emergence of the salt-and-pepper pattern continues until E4.0. The colour code is: *grey* for blastomers in which Nanog and Gata6 are low, *green* for ICM cells, *red* for Epi cells and *blue* for PrE cells. **b** Gene regulatory network (*GRN*) present in each of the modelled cells. **c** Equations of the GRN model, describing the rate of change of concentrations of Gata6 (*G*), Nanog (*N*), FgfR2 (*FR*), secreted Fgf4 (*Fs*) and perceived Fgf4 (*Fp*) as well as the level of activity of the Erk pathway *(ERK)*. Index *i* denotes the *i*th cell and *n*_*i*_ the number of neighbouring cells. See text for details. Definitions and values of parameters used in simulations can be found in Supplementary Tables S1–S3

In the embryo, ICM cells arise from two successive waves of differentiative (asymmetric) divisions. Indeed, during the 8- to 16- and the 16- to 32-cell divisions, single cells divide to give rise to two outer cells that both remain at the periphery of the embryo or to one inner cell and one outer cell. Inner cells form the prospective ICM cells. In this work, we focus on the possible outcome of ICM cells (Fig. 1a) and do not consider TE cells explicitly. Thus, we start from three to five cells, which is the usual range of the number of inner cells at E2.5, generated during the first differentiative division. They are called In1 cells. As the next round of division occurs, these cells give rise to six or ten inner cells. In addition, new ICM cells created by the asymmetric division of the peripheral TE cells (In2 cells) are introduced in the simulated system. The number of added In2 cells depends on the number of In1 cells and is adjusted to fit a total of 12 ICM cells (i.e., 2(#In1)+#In2 = 12). Indeed, the total number of ICM cells is regulated over the two cell divisions by compensation mechanisms leading to a similar number of ICM cells at the 32 cell stage.^29, 30^ We performed all simulations with both three and five In1 cells, and we verified that the behaviour of the model is robust to the changes of the proportion of In1 and In2 cells.

### Modelling the gene regulatory network

The GRN describing the interactions between the transcription factors Nanog and Gata6, together with the interplay between these factors, secreted Fgf4 and Fgf receptor 2 (FgfR2) is schematised in Fig. 1b. We showed in previous studies^7, 26^ that this model is able to recapitulate most of the experimental observations related to the specification of ICM cells performed on WT and mutant embryos in various conditions. We made, however, an adjustment to the simulated GRN: we now consider that Fgf4 secretion is inhibited by Gata6, instead of being stimulated by Nanog as assumed before. This assumption is supported by the existence of a Gata6 binding site on the Fgf4 promoter.^31^ As Nanog and Gata6 are mutually exclusive, Nanog activation and Gata6 inhibition of Fgf4 secretion are functionally equivalent and it is probable that both regulations coexist. However, as discussed below, a major contribution of Gata6-induced inhibition of Fgf4 secretion must be assumed to ensure the robustness of the specification process with respect to initial concentrations of Fgf4 and when modelling maternally deleted Fgf4 mutant embryos.^14, 15^ As the model predicts that this regulation is predominant during early development, only this negative feedback is considered in the model for the sake of simplicity.

Equations (1)–(4) in Fig. 1c provide a phenomenological description of the GRN regulating the interactions between the transcription factors in a single cell. The precise arrangement of terms corresponds to the only combination that can account for key observations.^26^ In contrast to our previous model where we simulated a static population of cells, we now introduce a function *f*(*t*) that multiplies Eqs. (1)–(4) to account for a progressive expression of the transcription factors, corresponding to their increasing rates of expression from the 8 cell stage.^9^ Function *f*(*t*) is a nonlinear function of time, the expression and the parameters of which are discussed in Supplementary Information (Section 4).

**Fig. 2:**
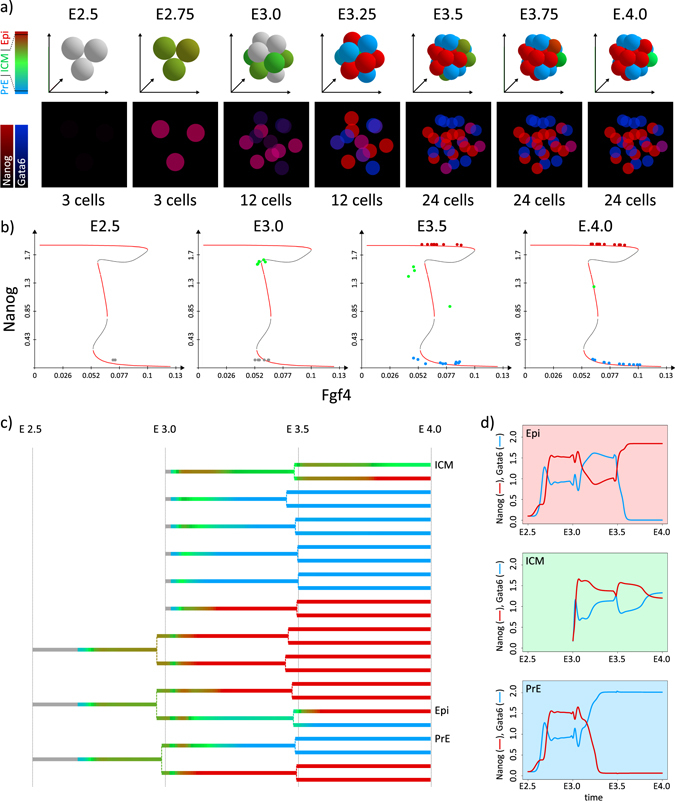
**a** Simulation snapshots showing the evolution of inner cells in the modelled embryo. Cells are coloured to show the proportion of the two main proteins—Nanog and Gata6, with *grey* indicating blastomers with very low (<1) Nanog and Gata6 concentrations. In addition, for easier comparison to the experimental data, below each 3D snapshot we show the levels of Nanog and Gata6 in individual cells in terms of fluorescence (2D projection). Corresponding embryonic times and cell counts (excluding TE cells) are indicated above and below each snapshot. In the colour scheme for 3D snapshots, *grey* indicates blastomers with very low Nanog and Gata6 concentrations, *green* denotes cells that have a comparable level of Nanog and Gata6 (ICM cells), *red* denotes high Nanog and low Gata6 concentrations characteristic for the Epi lineage and *blue* corresponds to cells having low Nanog and high Gata6 level, characteristic for the PrE lineage. In the model, we consider that a cell is Epi, if [Nanog] ≥ 10[Gata6], that it is PrE, if [Gata6] ≥ 10[Nanog] and ICM otherwise, as indicated on the colour code on the left. Parameter values and initial conditions are listed in Supplementary Tables S1– S3. **b** Fgf4/Nanog bifurcation diagrams showing stable (*red branche*) and unstable (*black branche*) stationary states of the system defined by Eqs. (1)–(4) (Fig. 1). Dots correspond to the cells of the embryo and are coloured according to their internal state. The diagrams correspond to embryo simulation in **a**. **c** Lineage tree of the embryo evolution showing the exact times of cell divisions and the proportion of Nanog and Gata6 (same colour code as in **a**). **d** Evolution of Nanog and Gata6 concentration in three examples—epiblast (*Epi*, *red background*), inner cell mass (*ICM*, *green background*) and primitive endoderm (*PrE, blue background*). The three example cells are indicated in the lineage tree in **c**

When considering Fgf4 concentration (*Fp*) as a control parameter, the model displays tristability in a range of values of *Fp*, as studied before^7, 26^ and shown in Fig. 2b. Each of the three stable steady states corresponds to one cell fate: a high Nanog/Gata6 ratio corresponds to the Epi state, whereas a high Gata6/Nanog ratio corresponds to the PrE state. The steady state corresponding to Nanog and Gata6 coexpression corresponds to the ICM state. Equation (5) describes the evolution of Fgf4 secreted by cell *i* in the extracellular medium. The concentration of Fgf4 perceived by cell *i* is computed as the average of the concentrations of Fgf4 secreted by itself and its *n*_*i*_ nearest neighbours. *J*_*i*_ stands for the set of neighbours of cell *i* in Eq. (6). Cells *i* and *j* are considered to be neighbours, if the distance *d* between their centers of mass satisfies the following condition:

$$d < {f_D}\left( {{r_i} + {r_j}} \right),$$

where *r*_*i*_ and *r*_*j*_ are their corresponding radii and *f*_*D*_ is a parameter that scales the range of cell to cell interaction through Fgf4 (taken equal to 1.2, see Supplementary Table S2). Results of the simulations are not sensitive to the exact value of this parameter (Supplementary Fig. S9).

Parameter *γ*_*i*_, appearing in Eq. (6) is chosen randomly within the interval [−*γ*, *γ*], where *γ* is fixed for a given simulation. It stands for possible local heterogeneities in Fgf4 concentration. We indeed reasoned that, due to the high level of compaction in the developing embryo, the concentration of extracellular Fgf4 resulting from the secretion from neighbouring cells does not immediately average. It is also in agreement with the observed high variability in the expression of Fgf4 reported by ref. 32. The number of neighbours *n*_*i*_ depends on the cell, and changes at each cell division because of rearrangements in the embryo. The values of *γ*_*i*_ are randomly reallocated at each cell division as daughter cells are not at the same location as the mother cell and thus evolve in a new environment.

Most values of parameters and initial conditions of the variables are taken from our previous studies.^7, 26^ The initial conditions of the variables of the GRN correspond to precocious ICM cells. The parameter values are such that, in the absence of intercellular communication, all cells evolve towards a mature ICM state, characterised by the coexpression of Gata6 and Nanog. Initial conditions are the same for In1 (starting at E2.5) and In2 cells (starting at E3.0), except for a higher level of activity of the Erk signalling pathway (variables *FR* and *Erk*) in In2 cells to account for the different ages of the two types of cells. The list of parameter values and initial conditions is given in Supplementary Tables S1–S3. Code is available on request.

## Results

### Specification of Epi and PrE cells arranged in a 3D salt-and pepper pattern

The simulations of the specification of ICM cells into Epi or PrE cells in a 3D framework, taking into account divisions and both proveniences of ICM cells, show that the proposed GRN is able to account for the specification process observed *in vivo* in conditions that are more realistic than in our previous approach. As shown in Fig. 2a, precocious ICM cells characterised by low levels of Nanog and Gata6 further coexpress these two transcription factors, divide and interact through extracellular Fgf4, finally giving rise to a mixed population of Epi and PrE cells. The timing of the progressive coexpression of Nanog and Gata6 followed by the mutual exclusion of the expression of these factors is in agreement with experimental observations.^7, 9, 18, 32^ Among the 24 final cells, 46 (±4) % adopt the Epi fate, 45 (±5) % adopt the PrE fate, whereas 10 (±7) % are still in the ICM state (100 simulations). Among the latter cells, some of them will specify later, whereas the remaining ‘undecided’ are likely suppressed by apoptosis in the embryo. The simulated proportions are in agreement with *in vivo* observations.^7^

Figure 2b shows the evolution of the cells in the (Fgf4, Nanog) plane, each cell being depicted by a *dot* whose colour represents the state of its internal GRN. The bifurcation curves indicate the steady states of Eqs. (1)–(4). From the precocious ICM state corresponding to E2.5, In1 cells increase their level of Nanog and Gata6 (*green dots* representing ICM cells at E3.0). At the same time, In2 ICM cells (*grey*) appear. Fgf4 is initially relatively high because of the maternal contribution and because of its synthesis by the zygote at a low average level of Gata6. Then, it decreases because it is metabolised and at the same time not yet significantly produced by the embryo. Following this decrease in Fgf4, some cells reach the Epi state (E3.5). The accompanying decrease in Gata6 in these cells then promotes Fgf4 secretion in the extracellular medium, which favours the specification of ICM cells into PrE cells. Once on their steady-state branch, Epi and PrE cells tend to remain in this state because of hysteresis.

More detailed views of the internal state evolution of the simulated cells are presented in the lineage tree and examples of time series are shown in Figs. 2c and d. After some latency, both Nanog and Gata6 increase together and the cells evolve towards the ICM state. From this stage on, the cells that perceive a bit less Fgf4 begin to produce Nanog faster and evolve towards the Epi state. Their level of Gata6 decreases and they secrete more Fgf4 and incite other cells to become PrE cells. It is also visible that each cell division is accompanied by some variations in the levels of Nanog and Gata6, due to changes in Erk signalling caused by the new environment of the daughter cells.

Thus, tristability allows for a self-regulated mechanism of ICM cells specification into Epi or PrE cells. The GRN and cell-to-cell interactions through Fgf4 signalling that has been validated earlier as a mechanism of Epi and PrE specification on a static two-dimensional population of 25 cells is robust when considering a more realistic evolution scheme. This includes the increase in the number of neighbours brought about by the consideration of the third dimension, cell division and the resulting changes in the environment of the individual cells, and the different proveniences of ICM cells.

### Statistical analysis of the destiny and characteristics of the simulated embryo

**Fig. 3:**
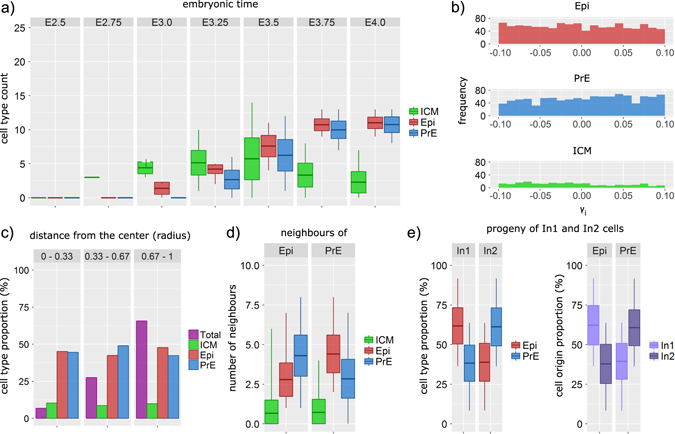
Analysis of the simulated embryos with the default values of parameters given in Supplementary Tables S1–S3. The analysis is done for a sample of 100 simulated embryos. **a** Evolution of ICM, Epi and PrE by cell count from E2.5 to E4.0. The *darker lines* in the *boxes* show the mean values, the *boxes* show the standard deviation, whereas the *vertical lines* indicate the minimal and maximal value of the sample. **b** Distribution of the final random parameter *γ*_*i*_ for each of the three cell fates—epiblast (*Epi, red*), primitive endoderm (*PrE, blue*) and inner cell mass (*ICM, green*) showing the absence of correlation between the final value of *γ*_*i*_ and the cell fate. **c** Spatial distribution of each cell type at various distances from the centre of the embryo. The distances are normalised by the radius of the embryo. The total number of cells (magenta) shows how many cells were observed at each layer compared with the number of cells in the whole embryo. **d** Number of neighbouring cells by type surrounding Epi (left) and PrE cells (right), showing that Epi cells are surrounded by more PrE cells than by cells of their own type, and vice versa. **e** On the *left hand side*—the proportions of Epi and PrE cells in In1 and In2 cell progeny. On the *right hand side*—the proportions of In1 and In2 originating cells in the final Epi and PrE cell population

The model can be used to perform a statistical analysis of the detailed characteristics of the embryo. Figure 3a shows the evolution of the number of ICM, Epi and PrE cells from E2.5 to E4.0. These proportions are consistent with experimental observations.^6, 18^ The simulation results also show that variations around average proportions are large, especially around E3.5. In average, Epi cells appear earlier than PrE cells (E3.18 vs. E3.38) in the model and in experiments.

In the simulations presented up to now, heterogeneity arises from the values assigned to parameter *γ*_*i*_, supposed to represent local inhomogeneities in extracellular Fgf4 concentration. We checked that this assumption does not bias the specification process. As shown in Fig. 3b, there is no significant correlation between the final value of *γ*_*i*_ and the fate of cell *i*. Indeed, for *γ*_*i*_ ∈ [−0.1,0.1] the average values of *γ*_*i*_ are −0.003 (±0.058), 0.006 (±0.057) and −0.033 (±0.057) for Epi, PrE and ICM cells, respectively. We next questioned the possible existence of some kind of positional information by computing the number of cells of each type in three different layers of the simulated embryo at E4.0 (Fig. 3c). The model predicts that all cell types are massively represented in all parts of the embryo. However, it is clear from Fig. 3d that Epi cells are preferentially surrounded by PrE cells and vice-versa, which is in line with the salt-and-pepper pattern observed before the cell sorting process leading to the blastocoel.

In the model, we consider that ICM cells arise from two successive waves of differentiative divisions by simulating two different initial populations of cells: initial In1 cells are present since the beginning of the simulations, whereas In2 cells are ‘added’ just after the division of the In1 cells, to represent the cells generated by the second round of differentiative divisions. Figure 3e shows that In1 cells have a preference to become Epi, whereas In2 cells have a preference to become PrE, in qualitative agreement with experimental observations.^30, 33^ However, as shown in Supplementary Fig. S4, the bias is quantitatively dependent on the respective numbers of In1 and In2 cells, which may explain the different conclusions reached by distinct experimental works.^17, 30, 34^

### Comparison with experimental data

**Fig. 4:**
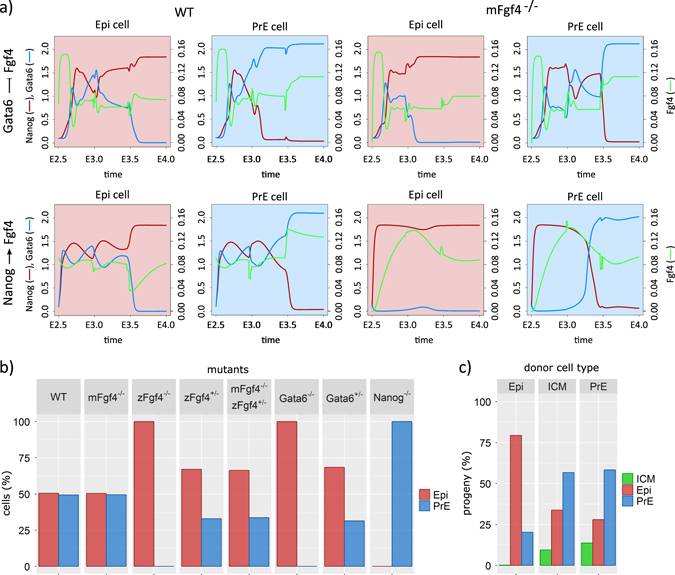
**a** Evolution of Nanog (*red curve*), Gata6 (*blue curve*) and Fgf4 (*green curve*) concentrations in an Epi and a PrE cell in wild type (*left*) and maternal Fgf4 mutant (*right*) with two hypotheses—the current model described in Fig. 1 (*top*) and the previous model described in refs. 7 and 26. In the current model, Fgf4 is inhibited by Gata6, whereas in the previous one it was activated by Nanog. **b** The proportion of epiblast (*Epi*) and primitive endoderm (*PrE*) progeny in different *in silico* mutants as indicated above the histograms. Mutants are simulated by setting the rates of synthesis of the appropriate compound to 0 for full mutants and to 75% of their default value for the heterozygous mutant to account for compensation.^7^ **c** Simulations of implantation experiments in which individual cells were taken at three different stages of the embryo development (E3.75, E3.75, E4.25) and implanted in host embryos at E2.5. Results obtained for the different ages of the donor embryos are pooled together. The three graphs show the composition of the progeny of the Epi, inner cell mass (*ICM*) and PrE donor cells

In vivo, the GRN and signalling pathways underlying the Epi vs. PrE cell specification can be further clarified through observations on mutant embryos and experiments in which the Fgf/Erk signalling pathway is exogenously manipulated. In particular, the phenotype resulting from zygotic and maternal/zygotic inactivation of Fgf4 has been investigated in ref. 14. These results can be used to infer the main mechanism involved in the regulation of Fgf4 secretion by Nanog and/or Gata6. Both the inhibition of Fgf4 secretion by Gata6 and the activation of Fgf4 secretion by Nanog represent plausible mechanisms. In the model, we have set the initial Fgf4 concentration to zero to simulate the maternal Fgf4 mutant (mFgf4^−/−^), and decreased the rate of Fgf4 production (*vsf* in Eq. (5)) to simulate the zygotic mutant. Figure 4a shows the evolution of Nanog, Gata6, and perceived Fgf4 (*Fp*) in a future Epi and a future PrE cell of simulated WT and mFgf4^−/−^ embryos following both assumptions related to the regulation of Fgf4 production. In WT (Fig. 4a, *left panels*), starting from a relatively high initial concentration of Fgf4 in line with experimental observations,^9, 15^ Fgf4 concentration initially increases in the case of the inhibition by Gata6. In contrast, in the case of the activation by Nanog, the level of Fgf4 remains approximately the same. In both cases, all cells coexpress Gata6 and Nanog before the specification as the level of Erk signalling is limited by the rather low level of expression of Fgf receptors, *FR* (Supplementary Figure S3). The predicted phenotypes of the maternal Fgf4 mutants are, however, drastically different for the two possible mechanisms. Although the effect of this mutation is very limited when it is assumed that Gata6 inhibits Fgf4 secretion, it prevents the passage through an ICM-like state in the reciprocal assumption (Fig. 4a, *right panels*). As the co-expression of Nanog and Gata6 around E3.5 is reported in mFgf4^−/−^ mice,^14^ we envisaged that the main regulation of Fgf4 secretion occurs because of its inhibition by Gata6, as formalised in Eq. (5). Moreover, we found that thanks to this change in regulation of Fgf4 synthesis, the cells always pass through the ICM state, whatever the initial conditions in Fgf4 concentration, which was not the case with the alternative regulation. This is the only modification of the modelled GRN with respect to previous versions.^7, 26^

As suggested by the similarity in the time evolutions of Nanog, Gata6 and Fgf4 in the simulated WT and mFgf4^−/−^ (Fig. 4a) mice, the model predicts that maternal Fgf4 has nearly no influence on the proportions of Epi and PrE cells at E4.0 (Fig. 4b). This is in agreement with observations reported in ref. 14, but not with those of ref. 15 that were obtained with a different mouse strain. By contrast, Fgf4 null mutant embryos exclusively comprise Nanog-expressing cells at the time of implantation.^14, 15^ In agreement with these results, all simulated cells evolve towards the Epi state when the rate of Fgf4 synthesis is set to 0 (Fig. 4b, zFgf4^−/−^). In the model, the heterozygous mutant (zFgf4^+/−^) displays proportions that are intermediate between the WT and the full mutant.

Results about Gata6 mutants are in agreement with those obtained previously with the static model.^7, 26^ Modelled Gata6 homozygous and heterozygous mutants reproduce populations of 100 and 70% of Epi cells at E4.0 (refs 7, 8), respectively, whereas Nanog mutants consist of 100% PrE cells.^6^ Besides affecting the proportions of the different populations, mutations also affect the timing of specification in the model. In the WT, PrE cells specify in average 4.7 h later than Epi cells. In the heterozygous Fgf4 mutant, this time interval increases up to 6.5 h, whereas it decreases to 3.4 h in the Gata6^+/−^ mutant (Supplementary Table S4).

As another test of the model, we simulated experiments of the implantation of donor Epi, ICM or PrE cells into recipient embryos. It was shown indeed that future PrE cells have a higher capacity to change fate than Epi ones.^35^ To test this, we simulated a wild-type developing embryo until different times (E3.25, E3.75, E4.25). Then every cell was considered as ‘a donor cell’, i.e., all their concentrations were saved. Every individual cell was then re-inserted in the center of a simulated ‘recipient embryo’ at E2.5 and its progeny at E4.5 was characterised. The analysis of the total progenies of all Epi, ICM and PrE donor cells from several donor embryos is shown in Fig. 4c. In agreement with experimental data,^35^ we found that Epi precursors display the lowest plasticity (Fig. 4c). It should be noted that, because we do not consider maturation processes, the model does not reproduce later loss of plasticity that involves the expression of additional transcription factors not included in the model. Interestingly, we could use the model to predict what would happen if donor cells were inserted in older recipient embryos (E3.75). In this case (Supplementary Fig. S5), the main difference is that the progeny of donor ICM and PrE cells would be made of ~75% of PrE cells, which is a higher proportion than when they are inserted in a younger recipient embryo.

**Fig. 5:**
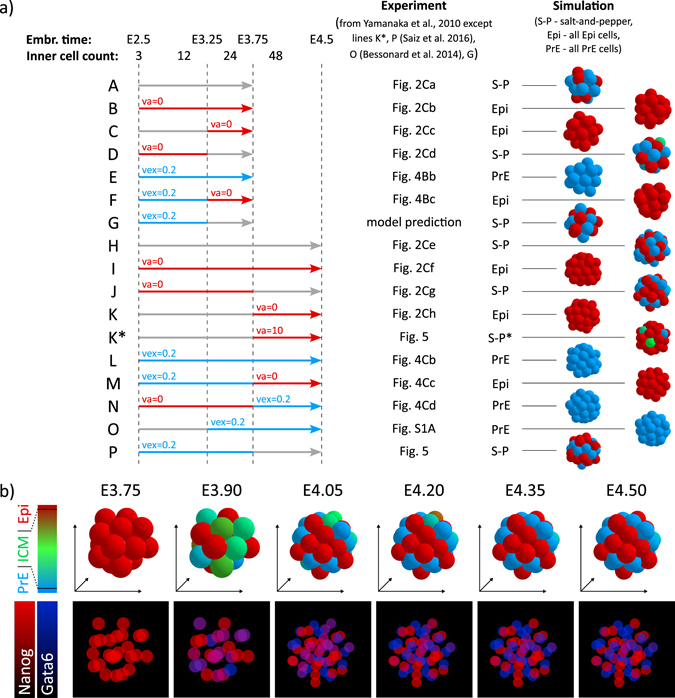
**a** Results of different *in silico* treatments. *Red lines* denote the periods of the Erk-pathway inhibition (*va* = 0, Eq. (3) in Fig. 1), whereas the *blue lines* denote the periods of addition of exogenous Fgf4 (*vex* = 0.2, Eq. (5) in Fig. 1). For each *in silico* experiment (A to P), we show the reference to the corresponding experiment, and the simulation outcome. Line K* corresponds to a partial inhibition of the Erk-pathway, i.e., *va* = 10. **b** Embryo evolution in the experiment J for the period from E3.75 to E4.5. The *top row* shows the embryo snapshots during the evolution, whereas the *bottom row* shows the levels of Nanog and Gata6 at the corresponding times

We showed in previous studies^7, 26^ that the model recapitulates an ensemble of observations performed on embryos when manipulating the Fgf/Erk signaling pathway. These experiments were simulated again with the present, more realistic model. Indeed, as time is now calibrated, we can distinguish between treatments of various durations. In addition, the realistic, dynamical scheme including cell division may alter the response of the model to external perturbations. Observations are summarised in Fig. 5. Simulations of the model including cell division allow for a detailed view of the evolution of the cells during and after these treatments. In particular, the model shows (Figs. 5aJ and b) that after removing Fgf/Erk inhibitors administrated from E2.5 to E3.75, cells rapidly start to co-express Nanog and Gata6 in a salt-and-pepper manner, as reported.^17, 18^ The numbers of cells of the various types at the end of this simulation are 21.5 ± 1.2 Epi, 20.7 ± 2.1 PrE and 5.8 ± 2.8 ICM. Simulations also predict that the level of Fgf/Erk inhibition has a strong consequence on the outcome of the treatment. Indeed, a full (Fig. 5aK) or a partial (Fig. 5aK*) inhibition of this pathway from E3.75 to E4.5 lead to an embryo containing only Epi cells or a mixed population of cells, respectively. This computational observation may explain, at least in part, why authors of ref. 17 who used a combination of two inhibitors reported only Nanog positive cells in these conditions, whereas authors of ref. 18 who used only one inhibitor reported a mixed population of cells.

### Robustness of the specification process with respect to cell division and investigation of the possible source of initial heterogeneity

**Fig. 6:**
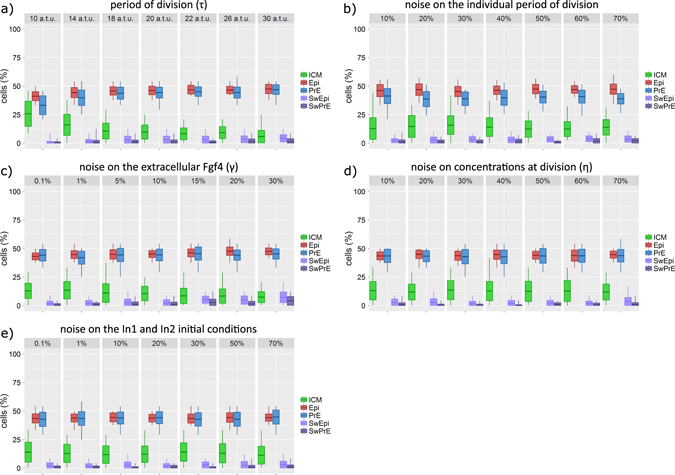
Analysis of embryo development sensitivity on: **a** the mean division period (*τ*), **b** the noise on the timing of individual cell divisions, **c** the noise on the extracellular Fgf4 (*γ*), **d** the noise on repartition of gene regulatory network (*GRN*) factors in daughter cells at division (*η*), **e** the noise on the initial conditions of Nanog, Gata6, Fgf receptors and Erk in both In1 and In2 cells. Each graph shows the mean cell count, standard deviation (*box*) and minimal and maximal cell count (*vertical line*) at E4.0 for inner cell mass (*ICM*), epiblast (*Epi*), primitive endoderm (*PrE*) cells and as well as for the number of switches from PrE to Epi (*SwEpi*) and from Epi to PrE (*SwPrE*). **a** and **b** show that the model is quite robust on the moderate changes in the cell division period and on the noise on the timing of individual cell divisions. A more detailed description of the analysis presented in this figure is given in section 5 of Supplementary Information

Sensitivity analyses confirm the robustness of the proposed specification mechanism (Supplementary Fig. S8 and Supplementary Table S5; see also Supplementary Fig. S4 of ref. 26): each parameter can be changed by ±10% without affecting the final proportions of the different cell types. After this validation, the model can be used to assess the effect of a change in the values of parameters not directly related to the GRN and that cannot easily be manipulated *in vivo*. We first investigated if the division period *τ* affects the proportions of each cell type. Figure 6a shows that shorter division times favour large proportions of ICM cells. In this case indeed, the respective timings of division and GRN evolution are modified in such a way that divisions stop before full specification in many cells of the embryo. Up to now, cellular divisions are synchronised among the whole embryo. Indeed, although all cells do not divide exactly at the same time, divisions are organised in rounds (i.e., all cells will have undergone their *n*th division at *nτ*). We next tested if the removal of this condition influences the outcome of the simulations. In Fig. 6b, each cell divides with its own periodicity *τ*_*i*_, with the average of the *τ*_*i*_’s being equal to *τ*. Interestingly, the proportions of each cell type are very robust towards cell-to-cell fluctuations on the division periods.

The specification of ICM cells into a mixed population of Epi and PrE cells arranged in a salt-and-pepper pattern implies some stochastic process, the exact nature of which remains to be determined. In our model, this source of noise is assumed to be related to some restriction in the process of Fgf4 diffusion due to the high level of compaction in the embryo (parameter γ_i_ in Eq. (6), Fig. 6c). We showed previously that this hypothesis is more likely than a primary role played by fluctuations in the expression levels of the transcription factors due to molecular noise.^26^ A third possibility, which can only be challenged with a model including cell division, would involve some random asymmetry in the repartition of the various compounds implied in the GRN during cell division, measured by parameter *η* defined above. The model predicts that indeed, in the absence of noise on extracellular Fgf4 (γ_i_ = 0), variations of the order of 10% in the repartition of molecules at division can account for the occurrence of the salt-and-pepper pattern, with appropriate proportions of Epi and PrE cells, although the number of ICM cells at E4.0 remains somewhat elevated, as shown in Fig. 6d. Moreover, in the simulations considering this source of noise even at high intensity, the number of switches between cell types remains very low in agreement with experimental observations.^18, 36^ Similarly, noise on the initial conditions of In1 and In2 cells (corresponding to slightly uneven repartition of molecules in the differentiative divisions) also suffices to account for the mixed population of Epi and PrE cells, with a slightly elevated number of ICM cells, as in the previous case (Fig. 6e). In the latter case, we checked that the fate of the progeny of the In1 and In2 cells is not biased by these initial conditions (Supplementary Fig. S6).

In conclusion, we found that the modelled specification process is rather robust with respect to external noise, whereas internal fluctuations in the numbers of molecules seem to have a constructive role in breaking the initial symmetry of identical ICM cells to initiate cell specification and the arrangement of the cells in a salt-and-pepper pattern. The noise on extracellular Fgf4 leads to less ICM cells at E4.0 than other sources of noise, which suggests that it is associated with a faster specification.

## Discussion

The emergence of a mixed population of Epi and PrE cells from a single population of ICM cells can be described by a simple and robust GRN based on interactions between Nanog, Gata6 and Erk signalling.^7, 26, 37^ Although mutual repression of Nanog and Gata6 is sufficient to reproduce the dynamics of ES cells transiently expressing Gata factors,^37^ this bistable model has not been able to explain the evolution from precocious to mature ICM cells co-expressing both transcription factors that is not an attracting, stable steady state in this situation. From a systemic point of view, the robust evolution towards the ICM state before Epi/PrE specification requires the existence of three stable steady states. The specification towards one of these fates is controlled by Fgf4, secreted in the extracellular medium by individual cells. The model proposed in this study provides a complete and self-autonomous 3D description of this process including cell division. Besides confirming the adequacy of the proposed GRN to describe Epi vs. PrE specification in realistic conditions, the model allowed us to gain important physiological information that was not available from previous studies, which did not take into account earlier events or cell divisions.

Concerning the spatio-temporal characteristics of the evolution towards the salt-and-pepper pattern, the model predicts that, despite a high level of variability in the cell arrangement, Epi cells are preferentially surrounded by PrE cells and vice-versa. In the simulations as in the experiments, cells arising from the second round of differentiative divisions have a larger tendency to become PrE than Epi. It should be stressed that this result is not related to any intrinsic difference between the cells arising from both rounds of division, but is due to differences in the timings of specification. Observations on mutant embryos and on WT ones submitted to manipulation of the Erk activity were also recapitulated by the model. Interestingly, to account for the fact that maternal Fgf4^−/−^ mutants initially coexpress Nanog and Gata6, the model now considers that the main regulation of Fgf4 secretion is the inhibition by Gata6, rather than the activation by Nanog as in our previous studies. As a last comparison with experimental data, we simulated the experiments of cell transfer from a donor to a recipient embryo, allowing to reproduce the fact that Epi cells appear to be much less plastic than other cell types. Interestingly, these results were obtained without considering additional factors related to cell commitment and thus shed light on a differential plasticity associated with the GRN itself. Our model also suggests that this observation may vary depending on the age of the recipient embryo.

The simulations emphasize that the proposed specification mechanism is very robust towards cell divisions, even if their timing is noisy. Moreover, the noise induced by an uneven distribution of the transcription factors at division may have a constructive role. Indeed, in the model, this noise can replace the noise on extracellular Fgf4 in the establishment of the salt-and-pepper pattern. This prediction contrasts with our previous conclusion that it is unlikely that intrinsic molecular noise represents the source of noise responsible for the salt-and-pepper pattern, as it increases the number of unrealistic switches between the Epi and PrE fates and drastically reduces the time spent by the cells on the ICM state.^26^ Here, it appears that the noise on the repartition of molecules at division has a much more limited effect, allowing sufficient noise to initiate specification but not cell fate switching. It is thus plausible that in the embryo, two sources of noise such as extracellular Fgf4 and slightly uneven divisions, are concomitantly at play, as suggested by ref. 32. However, it is unlikely that uneven divisions are the only source of heterogeneity. In Fgf4^−/−^ mutants indeed, sustained exogenous Fgf4 fails to rescue the salt-and-pepper pattern observed in the WT. Instead, embryos cultured in such conditions exhibit a unique cell type, the nature of which depends on the Fgf4 concentration.^14^

In the future, the present model should be extended to take explicitly into account the diffusion of extracellular Fgf4, as well as the expression of additional transcription factors related to cell fate commitment. In addition, a polarisation-induced specification of cells into TE^38^ and cell sorting from the salt-and-pepper pattern to the distinct PrE and Epi cell layers characterizing the late blastocyst^39^ could be modelled to get a global 3D computational representation of early mammalian development. These works will however require the consideration of complex biomechanical processes such as cell polarity and actomyosin contractility.^40^

## Electronic supplementary material

Supplemental Material
