# Supplementary material for: A multiscale model of early cell lineage specification including cell division
Source: NPJ Syst Biol Appl. 2017 Jun 9;3:16. doi: 10.1038/s41540-017-0017-0 (PMC5466652; doi:10.1038/s41540-017-0017-0)
Supplement: Supplementary file 1 — Supplemental Material [file 41540_2017_17_MOESM1_ESM.docx]

**Supplementary Information**

**A multiscale model of early cell lineage specification including cell division**

Alen Tosenberger, Didier Gonze, Sylvain Bessonnard,

Michel Cohen-Tannoudji, Claire Chazaud, Geneviève Dupont

**1. Definitions and values of the parameters**

| Parameter | Description | Value (a.u.) |
| --- | --- | --- |
| vsg1 | Maximum rate of Gata6 synthesis caused by ERK activation | 1.202 |
| vsg2 | Maximum rate of Gata6 synthesis caused by its auto-activation | 1 |
| vsn1 | Basal rate of Nanog synthesis | 0.856 |
| vsn2 | Maximum rate of Nanog synthesis caused by its auto-activation | 1 |
| vsfr1 | Basal rate of Fgfr2 synthesis | 2.8 |
| vsfr2 | Maximum rate of Fgfr2 synthesis caused by Gata6 activation | 2.8 |
| vsf | Maximum rate of Fgf4 synthesis | 0.3 |
| va | ERK activation rate | 20 |
| vin | ERK inactivation rate | 3.3 |
| kdg | Gata6 degradation rate | 1 |
| kdn | Nanog degradation rate | 1 |
| kdfr | Fgfr2 degradation rate | 1 |
| kdf | Fgf4 degradation rate | 2 |
| Kag1 | Threshold constant for the activation of Gata6 synthesis by ERK | 0.28 |
| Kag2 | Threshold constant for Gata6 auto-activation | 0.55 |
| Kan | Threshold constant for Nanog auto-activation | 0.55 |
| Kafr | Threshold constant for the activation of Fgfr2 synthesis by Gata6 | 0.5 |
| Kaf | Threshold constant for the inhibition of Fgf4 synthesis by Gata6 | 0.75 |
| Kig | Threshold constant for the inhibition of Gata6 synthesis by Nanog | 2 |
| Kin1 | Threshold constant for the inhibition of Nanog synthesis by ERK | 0.28 |
| Kin2 | Threshold constant for the inhibition of Nanog synthesis by Gata6 | 2 |
| Kifr | Threshold constant for the inhibition of Fgfr2 synthesis by Nanog | 0.5 |
| Ka | Michaelis constant for activation of the ERK pathway | 0.7 |
| Kin | Michaelis constant for inactivation of the ERK pathway | 0.7 |
| Kd | Michaelis constant for activation of the ERK pathway by Fgf4 | 2 |
| vex | Rate of addition of exogenous Fgf4 (in treatments) | 0.2 |
| r | Hill coefficient for the activation of Gata6 synthesis by ERK | 3 |
| s | Hill coefficient for Gata6 auto- activation | 4 |
| q | Hill coefficient for the inhibition of Gata6 synthesis by Nanog | 4 |
| u | Hill coefficient for the inhibition of Nanog synthesis by ERK | 3 |
| v | Hill coefficient for Nanog auto-activation | 4 |
| w | Hill coefficient for the inhibition of Nanog synthesis by Gata6 | 4 |
| x | Hill coefficient for the inhibition of Fgfr2 synthesis by Nanog | 1 |
| y | Hill coefficient for the activation of Fgfr2 synthesis by Gata6 | 1 |
| z | Hill coefficient for the activation of Fgf4 synthesis by Nanog | 4 |

Table S1: Definitions and values of the parameters of the GRN formalized by equations (1)-(6) in Figure 1c. Values are expressed in arbitrary units (a.u) and taken from previous work^7,26^ .

| Parameter | Description | Value |
| --- | --- | --- |
| r0 | Initial cell radius | 0.4 |
| m0 | Initial cell mass | 10E-06 |
| k_F_ | Strength coefficient of intercellular force function | 10E+07 |
| k_A_ | Relative cut-off distance of intercellular force function | 1.8 |
| c | Dampening coefficient for cell movement | 10E+04 |
| dt | Time step | 0.001* |
| τ | Division period | 20* |
| δ | Coefficient for the period of asynchronous cell division | 0.1* |
| γ | Maximal noise on perceived Fgf4 | 0.1 |
| t_add_ | Time of adding cells from second asymmetric division | 20 |
| f_D_ | Coefficient scaling cell to cell interaction through Fgf4 | 1.2 |
| f_max_ | Maximal rate for *f(t)* | 3 |
| t_1/2_ | Threshold constant for *f(t)* | 0.2 |
| cs1 | Critical level of Nanog or Gata6 to define an ICM state | 1 |
| cs2 | Critical ratio of Nanog and Gata6 to define Epi and PrE states | 0.1 |

Table S2: Values of the parameters characterizing the mechanical forces between cells, the cell division, the dynamics and the cell states. *For time units: 1 a.u. = 0.025 day.

| Initial conditions | In1 cells |  | In2 cells |
| --- | --- | --- | --- |
| G_i_ | 0.1 |  | 0.1 |
| N_i_ | 0.1 |  | 0.1 |
| FR_i_ | 1 |  | 2.8 |
| Erk_i_ | 0.1 |  | 0.25 |
| Fs_i_ | 0.066 |  | 0.066 |

**Table S3:** Initial conditions for cells originating from the first (**In1**) and the second (**In2**) differentiative division**.** Initial conditions for In2 cells are taken from ref. 7. Those for In1 cells are chosen to correspond to more precocious cells, with a lower level of expression of FfgfR2 receptors and Erk signalling.

**2. Model of interaction between cells**

We use a spherical model of cells, where each cell $i$ is defined by its radius $r_{i}$, mass $m_{i}$ and the position of its center of mass $\vec{p}_{i}$. To model physical interactions between cells $i$ and $j$ in a certain proximity of each other we introduce a function $\vec{F}_{ij}$ describing the potential and adhesive forces acting from cell $i$ on cell $j$:

$$\vec{F}_{ij}=\left\{ \begin{matrix} k_{F}\left( \frac{h_{0}}{h}-1 \right)\left( \frac{k_{A}h_{0}}{h}-1 \right)\frac{\vec{p}_{ij}}{h}, & h<k_{A}h_{0} \\ 0, & h\geq k_{A}h_{0} \end{matrix} \right.$$

where $k_{F}$ is the strength coefficient of the force, $k_{A}$ defines the range of influence of the adhesive force, $h_{0}=r_{i}+r_{j}$ is the sum of radii of the two cells, $\vec{p}_{ij}=\vec{p}_{i}-\vec{p}_{j}$ is the vector from the position of cell $j$ to the position of the cell $i$, and $h=\left| \vec{p}_{ij} \right|$ is the distance between their positions. The force function $\left| \vec{F}_{ij} \right|$ is depicted in Figure S1. The domain of the force function can be separated on three parts on which the force acts in different ways. If the two cells are at a distance less than the sum of their radii $h_{0},$there will be a repulsive force between them. If the distance is greater than $h_{0}$ but less than $k_{A}h_{0}$ there will be an attractive force between them. While if the distance between them is greater than $k_{A}h_{0}$, no force will apply to the cells^27,28^ .

Equations of cell motion follow from

$$m_{i}\frac{\vec{v}_{i}}{dt}+c\vec{v}_{i}=\sum_{j\neq i} \vec{F}_{ji},$$

where $m_{i}$ is the mass of the cell $i$, $\vec{v}_{i}$ is the velocity of the cell $i$, and $c$ is the damping coefficient (viscosity).


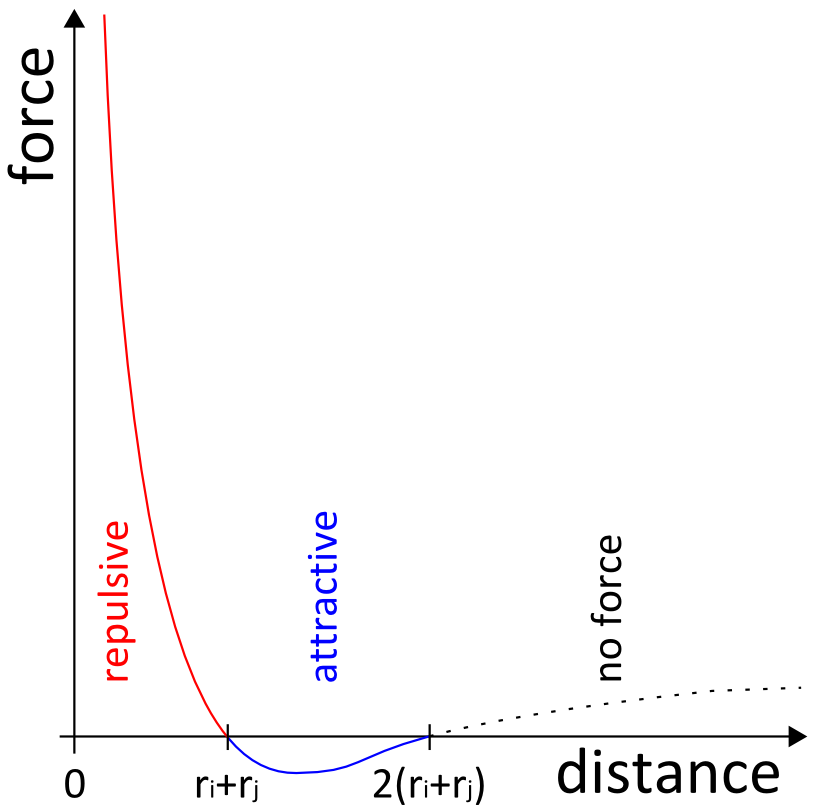


Figure S1: A schematic representation of the pair-wise force function used in the simulations, showing the intervals of repulsive force (red line), attractive force (blue line) and no force (black dotted line).

**3. Cell division**

At the cell division the mother cell $i$ is removed and two new daughter cells $j$ and $k$ are created instead. The plane of division is chosen randomly at every cell division and is defined by its normal vector $\vec{n}$. The initial positions of the two daughter cells are given by:

$$\vec{p}_{j}=\vec{p}_{k}=\vec{p}_{i}\pm\frac{r_{i}}{2}\vec{n}.$$

The mass and the volume of the two daughter cells are equal to the one half of the mass and of the volume mother cell. Then the radii of the daughter cells are given by:

$r_{j}=r_{k}=\frac{1}{\sqrt[3]{2}}r_{i}$.

Because of the interactions between cells, daughter cells are distant by 0.6 cell diameter in average after division. The concentrations of the GRN factors are preserved in the process of division and divided equally between the daughter cells in most simulations. This means that, as volumes of the daughter cells are decreased by half compared to the mother cell, the concentrations of factors will remain the same as in the mother cell.

**4. Function** $\boldsymbol{f}\boldsymbol{(t)}$ **describing the progressive expression of transcription factors**

To describe the evolution from the precocious ICM stage we use a non-linear evolution of the rates of synthesis of Nanog, Gata6 and of the compounds related to Fgf4 signalling in equations (1)-(4). We defined $f(t)$ as

$$f\left( t \right)=f_{max}\frac{t^{2}}{t_{1/2}^{2}+t^{2}}$$

Where *t* is the normalized time, i.e. corresponds to the time in a.u. divided by 60, as the whole simulation time corresponds to 60 a.u. (or 1.5 days), *t_1/2_* is the normalized time at which the rates are half-maximal. We found that a good agreement with *in vivo* observations was obtained if $f_{max}$= 3 and *t_1/2_* corresponds to E2.8. However, the final proportion of Epi and PrE cells is not very sensitive to the exact shape of the function $f(t)$.


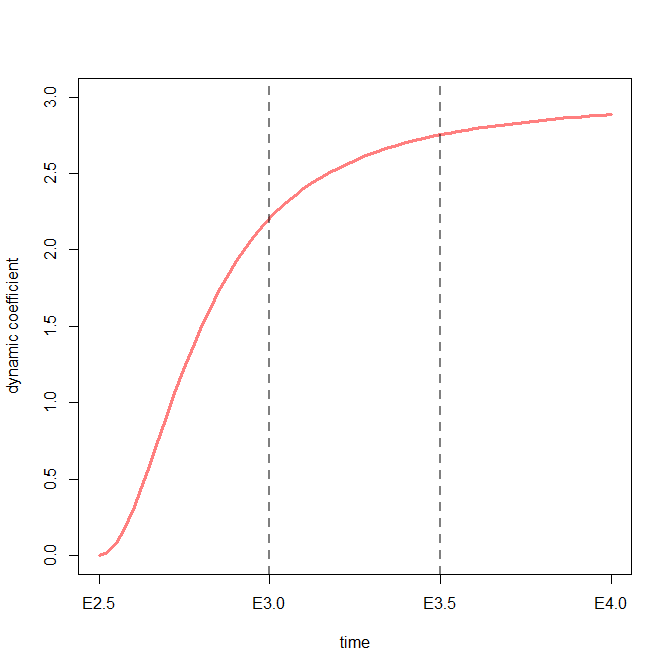


Figure S2: Function $\boldsymbol{f}\boldsymbol{(t)}$ used to describe the progressive expression of transcription factors.

**5. Noise analysis presented in Figure 6**

For every value of the parameters tested in Figure 6 the corresponding analysis was done on a sample of 50 simulated embryos. In **a)** we test the sensitivity of the embryo specification on the division period (τ). Division period was varied from 10 to 30 a.u., where 20 a.u. represents the default value (see Table S2). 1 a.u. corresponds to 0.025 days. For each value of τ we looked at the cell populations at time 3$\boldsymbol{\tau}$ a.u. For each value of τ, the darker horizontal lines show the mean values, the boxes represent the standard deviation around the mean, and vertical lines span from the minimal to the maximal value. The size of ICM population is shown in green, Epi in red and PrE in blue. In light purple we denote the number of cells which switched from PrE to Epi state, while in dark purple we denote the opposite. **b**) Analysis of the impact of cell divisions on the final populations. Instead of fixed cell division rounds, we considered the default value of τ as the mean period of division and introduced a noise parameter δ. For each value of δ, every cell would divide randomly in the period of $\tau\pm\delta/2$ measured from the time of its creation. Proportions of cell population in the final embryo are not affected by the noise on the period of cell division. **c)** Analysis of variation of noise on the extracellular Fgf4 (γ). The values of γ were varied from 0.1% to 30%, where 10% represents the default value. For very low noise it becomes harder for the ICM cells to specify to Epi or PrE cells, leading to a high ICM population at E4.0. As at each cell division the two daughter cells obtain a new value of $\gamma_{i}$, at very high level of γ the cells are more easily swayed to switch fates, either from Epi to PrE, or vice versa. **d)** As a possible alternate source of heterogeneity in the model we have substituted the noise on the extracellular Fgf4 (γ) by a noise on distribution of factors at the time of division (η), expressed in percent. At each cell division the concentrations of factors in mother cell are ascribed to the two daughter cells in a ratio $\eta_{i}:(1-\eta_{i})$, where $\eta_{i}$ is a randomly chosen value at the time of division from the interval $\left[ 0,\eta\right]$. In **e)** we test the possibility to substitute γ with the noise on the initial concentrations of Nanog, Gata6, Fgf receptors and Erk in both In1 and In2 cells. For every amplitude of noise μ expressed in percentage, the initial concentration of each factor for each cell was taken from the interval $\left[ 1-\mu, 1+\mu\right]$ of their corresponding default initial condition given in Table S3. The analysis shows that even small differences in initial concentrations (at E2.5) between cells can lead to the salt-and-pepper pattern. However, in both d) and e) we can observe a higher number of unspecified cells at E4.0 compared to the case c) where we apply noise on the extracellular Fgf4 concentration.

**6. Supplementary figures and tables**


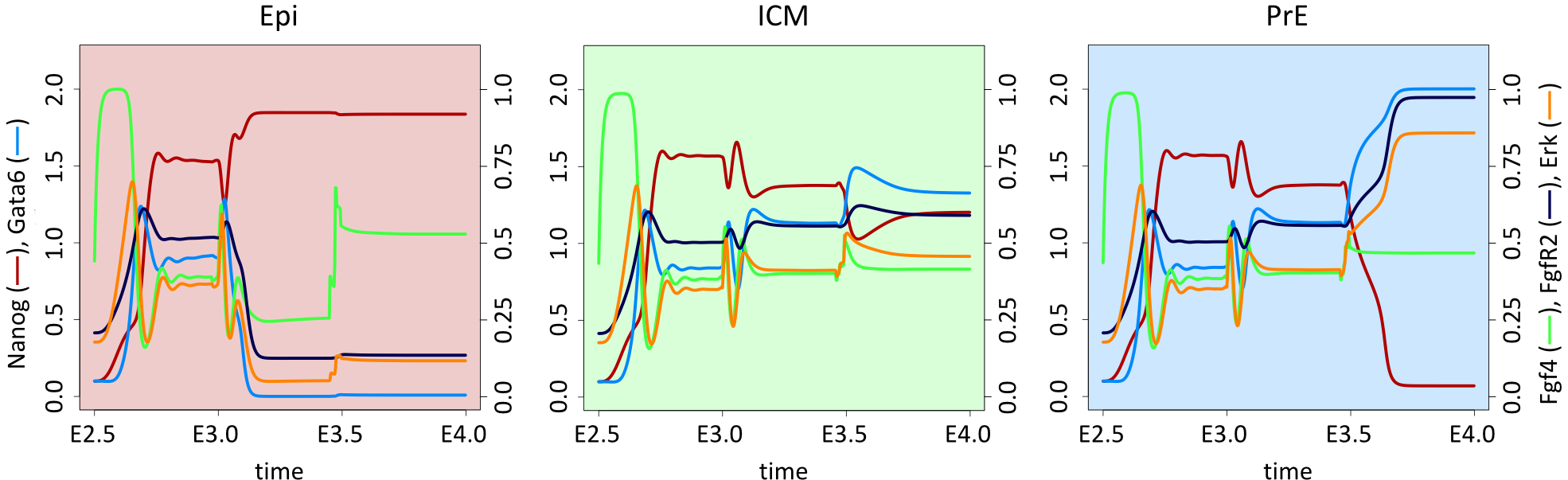


Figure S3: Temporal evolution of the variables of the model describing the GRN (equations 1 to 6 in Figure 1c). Typical examples of an Epi, an ICM and a PrE cell taken from a simulated wild type embryo. Nanog and Gata6 concentrations are shown in their simulated scale (left-hand side vertical axis), while the concentrations of Fgf4, FgfR2 and ERK are shown on a normalised scale (right-hand side vertical axis). The maximal values of Fgf4, FgfR2 and Erk equal 0.15, 4.83 and 0.57, respectively.


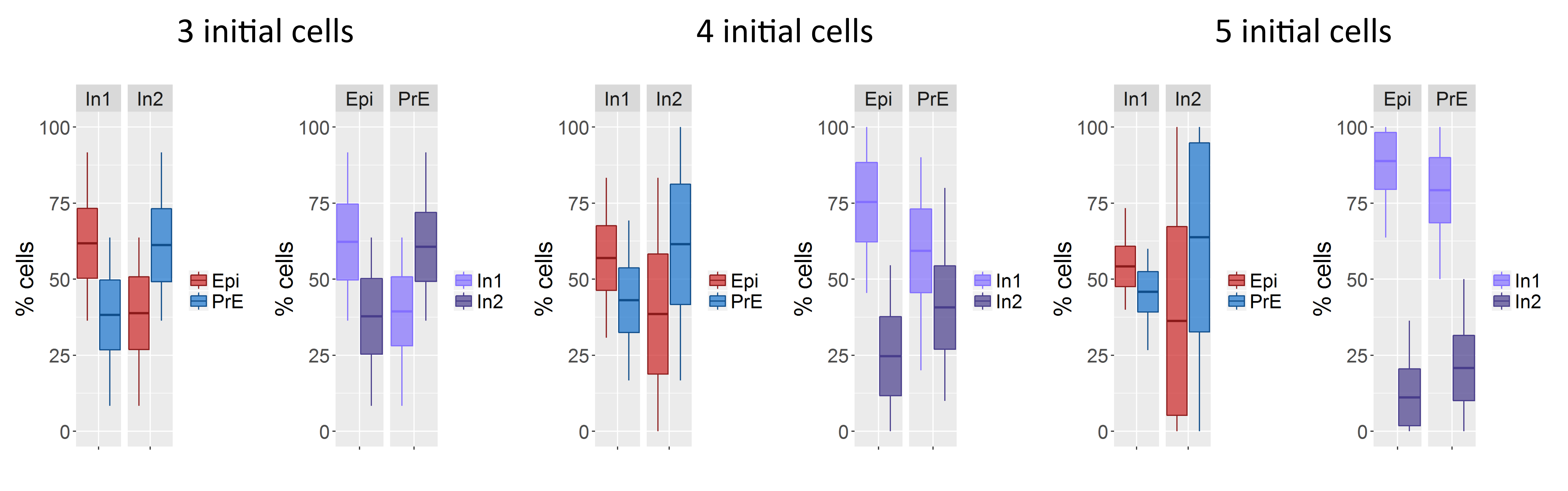


Figure S4: Effect of the number of In1 cells on the progeny of In1 and In2 cells. In1 and In2 progeny in case of 3 (left), 4 (middle) and 5 (right) In1 cells obtained as for Figure 3e of the main text.


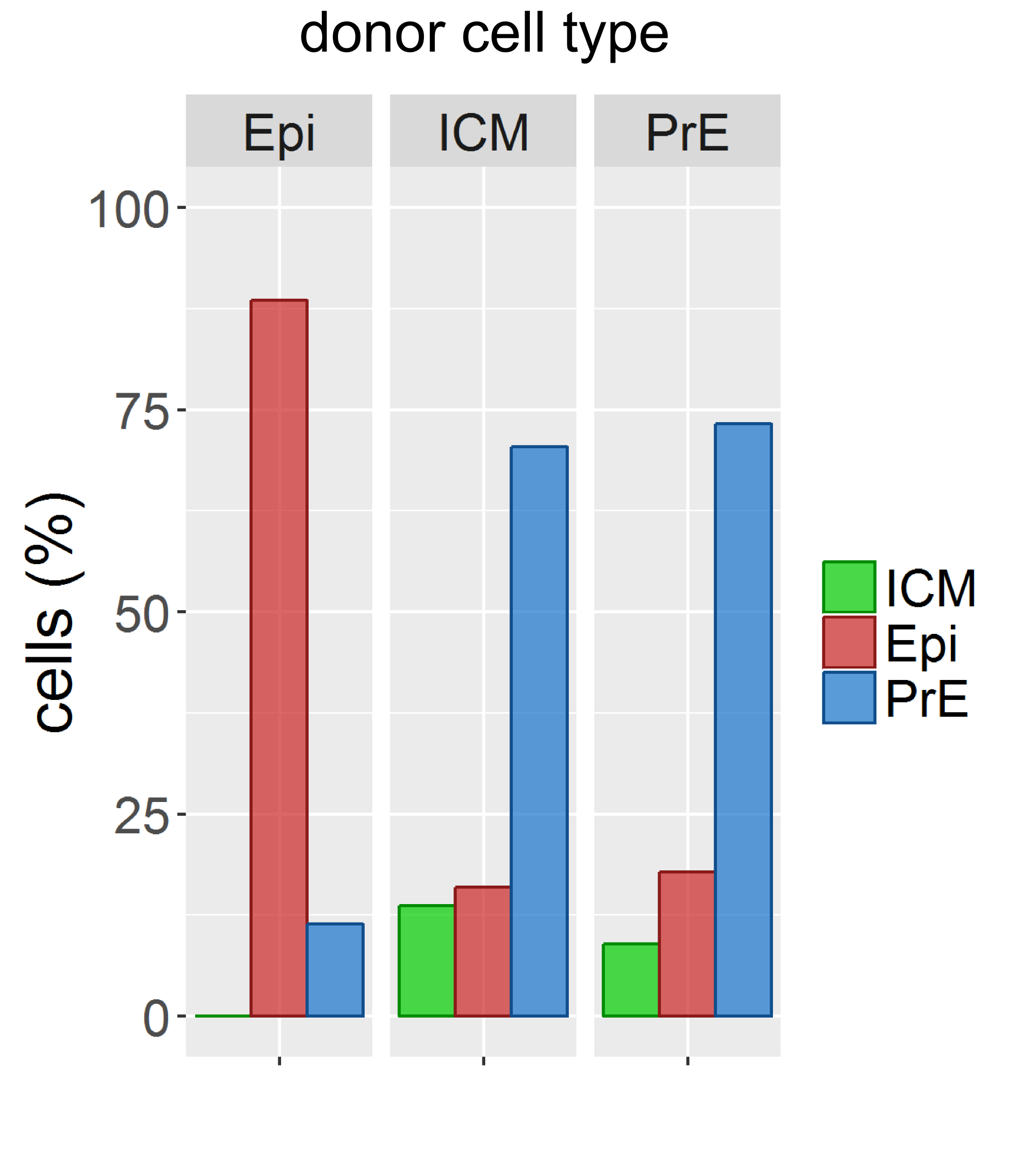


Figure S5: Simulations of implantation experiments in which individual cells were taken at different stages of the embryo development (E3.75, E3.75, E4.25) and implanted in host embryos at E3.75. The 3 graphs show the composition of the progeny of the Epi, ICM and PrE donor cells, respectively. As compared to the same simulations where the host embryos are younger (Figure 4c), both ICM and PrE cells have a much larger tendency to give rise to a PrE cell.


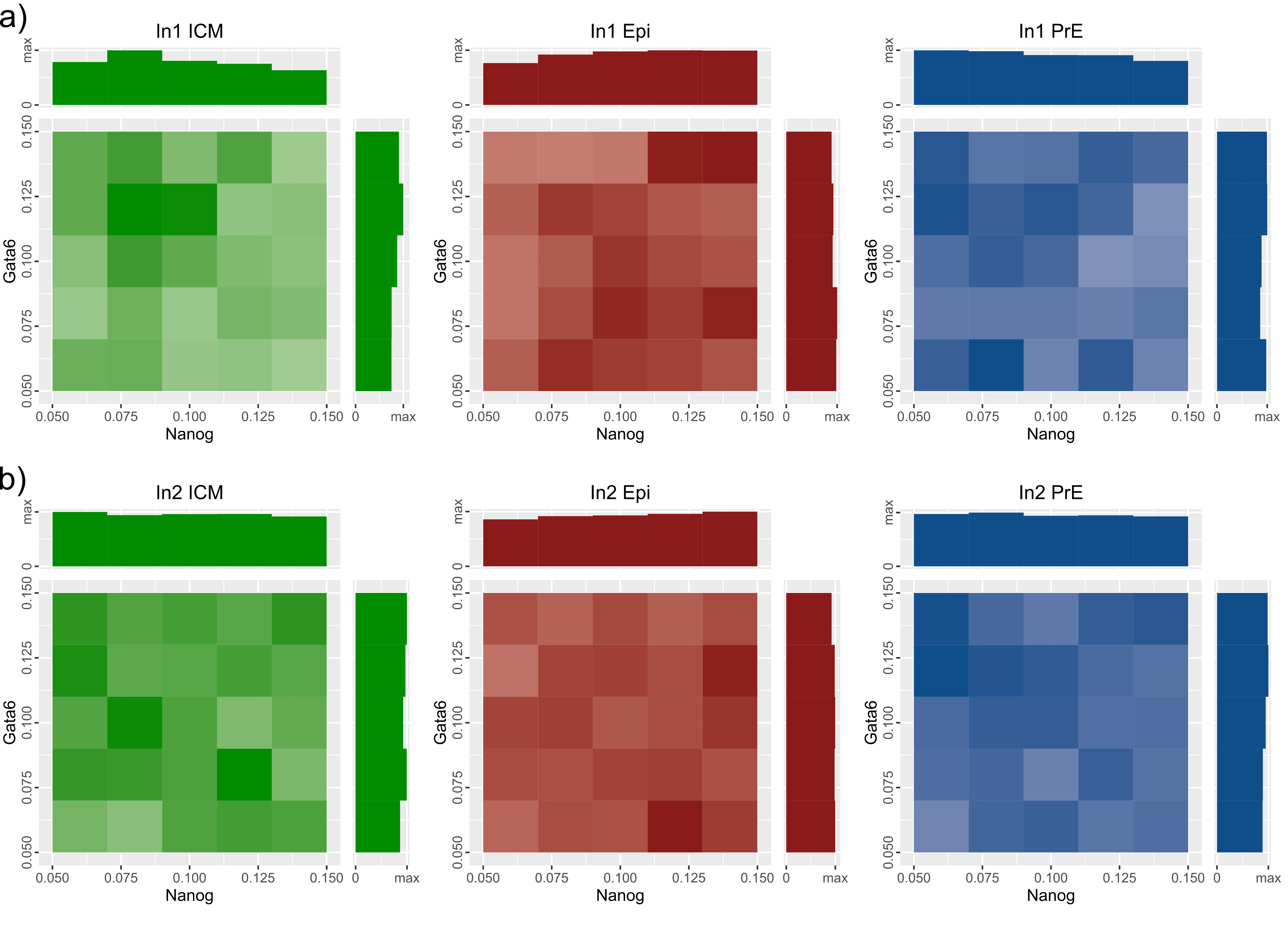


Figure S6: Robustness of cell fate with respect to initial conditions of In1 and In2 cells. Noise is applied to the initial conditions of all variables, except Fgf4 ($\boldsymbol{Fs}_{\boldsymbol{i}}\boldsymbol{)}$. Hence, for each cell the initial conditions of $\boldsymbol{G}_{\boldsymbol{i}}$, $\boldsymbol{N}_{\boldsymbol{i}}$,$\boldsymbol{FR}_{\boldsymbol{i}}$ and$\boldsymbol{ERK}_{\boldsymbol{i}}$ are randomly taken in the intervals [-50%,+50%] of their corresponding mean values indicated in the Table S3. Graphs in a) and b) show the dependence of cell fate on Nanog and Gata6 initial conditions in In1 and In2 cells respectively. The axes indicate the initial conditions of Nanog and Gata6, and colours indicate the frequency at which such a cell becomes an Epi (left), ICM (middle) or PrE (right) cell. The darker the colour, the higher the frequency, with white colour indicating 0 occurrences. The two histograms (above and to the right of each graph) represent the sum for a given Nanog and Gata6 initial concentration, respectively. We observe only a slight statistical tendency of cells to adopt Epi and PrE fate based on their initial level of Nanog and Gata6, but we can conclude that generally the final fate of the cell is not determined by its initial conditions.


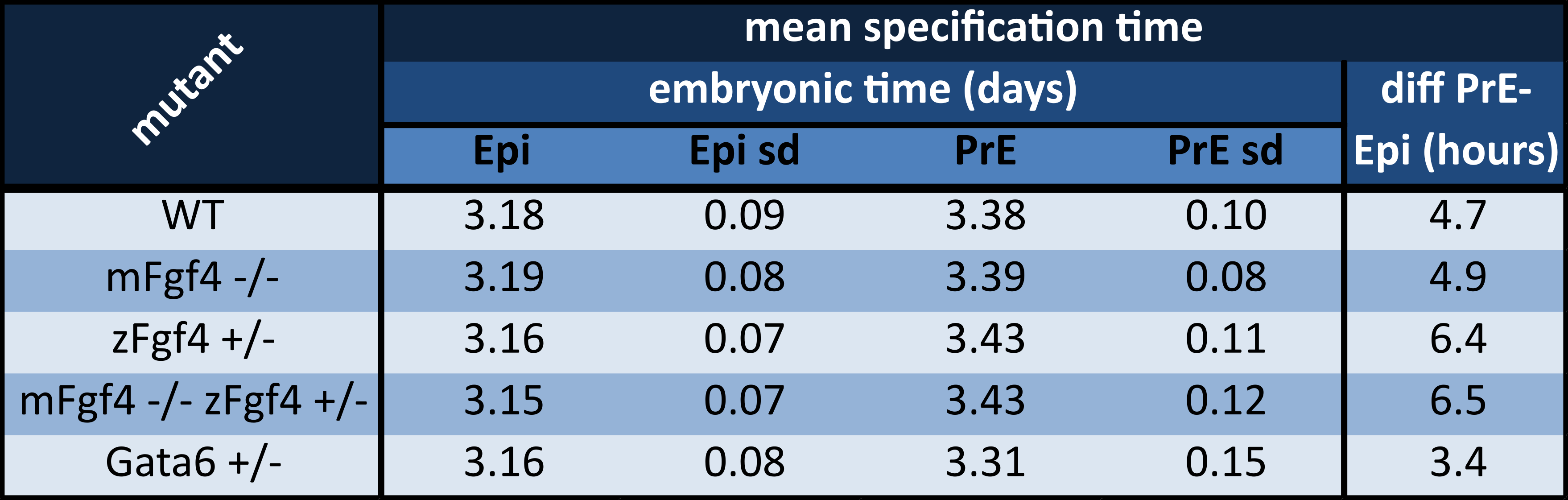


**Table S4:** Mean specification times of Epi and PrE populations in simulated WT and mutant embryos.


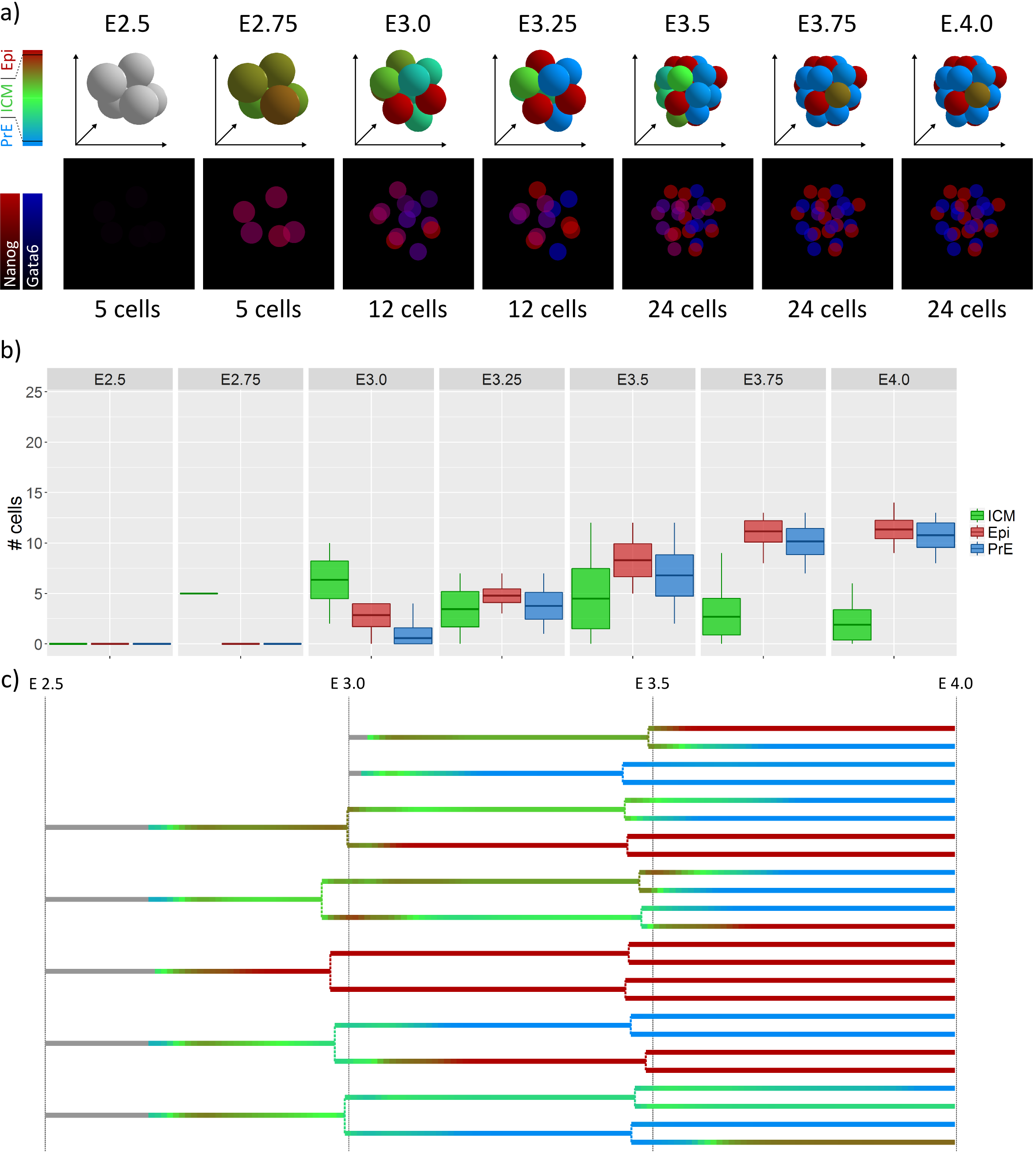


**Figure S7:** Simulations performed with 5 In2 cells and 2 In2 cells (instead of 3 In1 cells and 6 In2 cells). The relative numbers of cells originating from the 2 rounds of differentiative divisions do not impact on the behaviour of the model. Except for the numbers of In1 and In2 cells, all equations and parameter values are the same as in Figure 2.


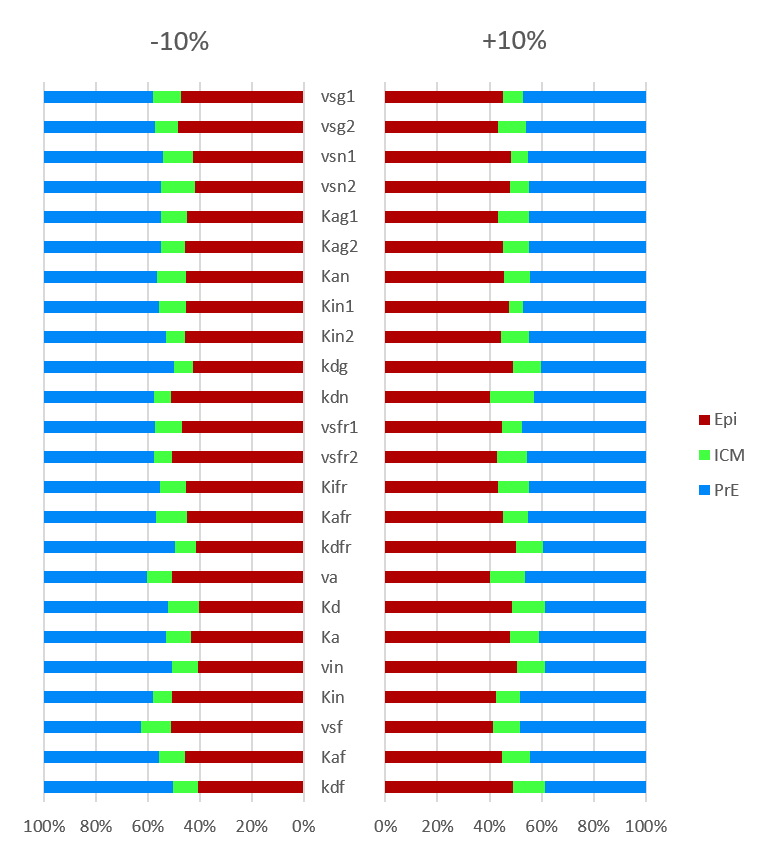


**Figure S8:** Parameter sensitivity analysis. Individual parameters were varied by +/- 10% of their corresponding default values (see Table S1). For each individual case 20 simulations were run and the mean percentage of ICM, Epi and PrE cells was calculated. In all cases we obtain salt and pepper pattern. The exact proportions of ICM Epi and PrE cell populations are given in Table S5.

| **-10%** | | | **GRN** | **+10%** | | |
| --- | --- | --- | --- | --- | --- | --- |
| **Epi** | **ICM** | **PrE** | **parameter** | **Epi** | **ICM** | **PrE** |
| 47% | 11% | 42% | **vsg1** | 45% | 8% | 47% |
| 49% | 9% | 43% | **vsg2** | 43% | 11% | 46% |
| 43% | 11% | 46% | **vsn1** | 48% | 7% | 45% |
| 42% | 13% | 45% | **vsn2** | 48% | 8% | 45% |
| 45% | 10% | 45% | **Kag1** | 43% | 12% | 45% |
| 46% | 9% | 45% | **Kag2** | 45% | 10% | 45% |
| 45% | 11% | 44% | **Kan** | 46% | 10% | 45% |
| 45% | 10% | 44% | **Kin1** | 48% | 5% | 47% |
| 46% | 7% | 47% | **Kin2** | 44% | 11% | 45% |
| 43% | 7% | 50% | **kdg** | 49% | 11% | 40% |
| 51% | 6% | 42% | **kdn** | 40% | 17% | 43% |
| 47% | 11% | 43% | **vsfr1** | 45% | 8% | 48% |
| 51% | 7% | 42% | **vsfr2** | 43% | 12% | 45% |
| 45% | 10% | 45% | **Kifr** | 43% | 12% | 45% |
| 45% | 12% | 43% | **Kafr** | 45% | 10% | 45% |
| 41% | 8% | 50% | **kdfr** | 50% | 10% | 39% |
| 51% | 9% | 40% | **va** | 40% | 13% | 46% |
| 40% | 12% | 48% | **Kd** | 49% | 13% | 39% |
| 44% | 9% | 47% | **Ka** | 48% | 11% | 41% |
| 41% | 10% | 49% | **vin** | 51% | 11% | 39% |
| 51% | 7% | 42% | **Kin** | 43% | 9% | 48% |
| 51% | 11% | 38% | **vsf** | 41% | 10% | 48% |
| 46% | 10% | 44% | **Kaf** | 45% | 11% | 45% |
| 41% | 9% | 50% | **kdf** | 49% | 13% | 39% |

**Table S5:** Influence of changes of individual GRN parameters on the ICM, Epi and PrE population.


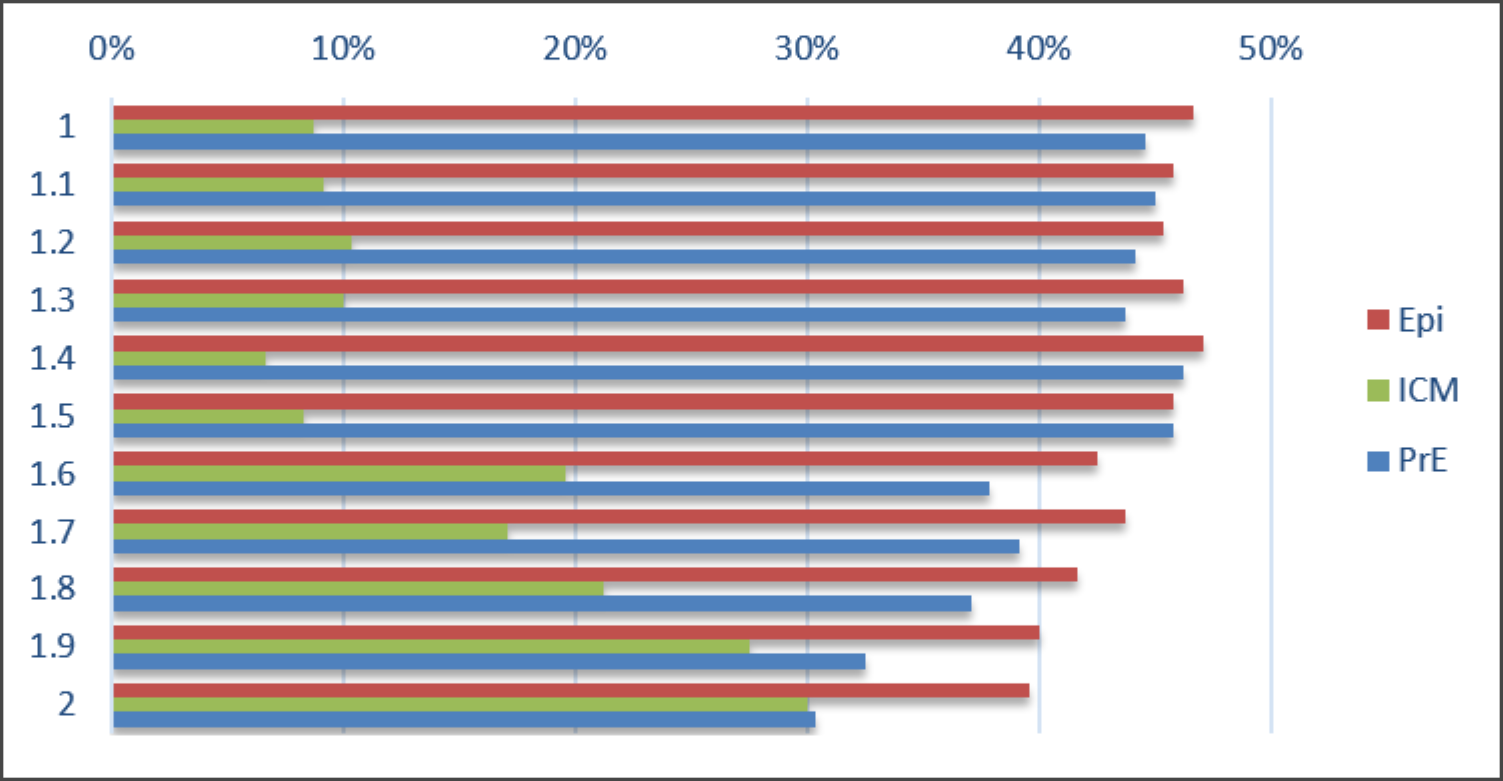


**Figure S9**: Sensitivity analysis of the model with respect to parameter f_D_ that scales the distance on which cells interact through extracellular Fgf4 (see section 2.3 of the main text). Values on the left are the values of f_D_ and bars indicate the proportions of the different cell types at the end of the simulation. The analysis was done on a sample of 50 simulations with the default values of the parameters listed in Tables S1, S2 and S3.
